# Coats disease

**DOI:** 10.11604/pamj.2015.20.205.6390

**Published:** 2015-03-06

**Authors:** Samar Younes, Hicham Tahri

**Affiliations:** 1 Ophtalmology Department, CHU Hassan II, Fez, Morocco

**Keywords:** Coats, aneurysms, laser photocoagulation

## Image in medicine

Coats’ disease is an idiopathic ophthalmic condition caused by a defect in the development of retinal vasculature, characterized by retinal telangiectasis, haemorrhages, intraretinal and subretinal exudation. There are two pathological processes, which are evident in Coats’ disease. The first consists of a breakdown of the blood-retinal barrier at the endothelial level, which causes plasma leakage into the vessel wall and thickening of parts of the vessel wall, becoming necrotic and disorganized. The second concerns the presence of abnormal pericytes and endothelial cells in retinal blood vessels, which subsequently degenerate, causing abnormal retinal vasculature and formation of aneurysms, as well as closure of vessels, leading to ischaemia. Here are several treatment modalities, especially laser photocoagulation and cryotherapy for mild to moderate stages of the disease as well as vitrectomy for advanced stages. Today, anti-VEGF agents are used as adjuvant therapy to other treatment options.

**Figure 1:**
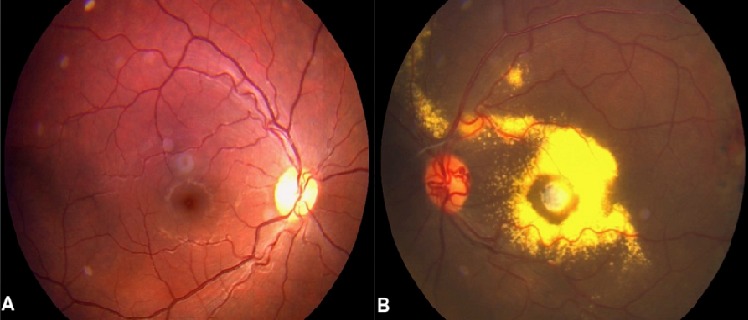
Colour fundus photo of a 21-year-old male patient presenting with Coats’ disease. In the figure, exudates and telangiectasis are present as well as dilation of retinal vessels, confirming Coats’ disease. Visual loss related to marked submacular lipid exudation and fibrosis

